# Reliability and Availability Evaluation of Wireless Sensor Networks for Industrial Applications

**DOI:** 10.3390/s120100806

**Published:** 2012-01-12

**Authors:** Ivanovitch Silva, Luiz Affonso Guedes, Paulo Portugal, Francisco Vasques

**Affiliations:** 1 Department of Computer Engineering and Automation, Federal University of Rio Grande do Norte, Campus Universitário 59078-900, Natal, Brazil; E-Mail: affonso@dca.ufrn.br; 2 ISR, Department of Electrical and Computer Engineering, University of Porto, Porto 4200-465, Portugal; E-Mail: pportugal@fe.up.pt; 3 IDMEC, Department of Mechanical Engineering, University of Porto, Porto 4200-465, Portugal; E-Mail: vasques@fe.up.pt

**Keywords:** dependability evaluation, wireless sensor networks, fault tree analysis, WirelessHART, ISA 100.11a

## Abstract

Wireless Sensor Networks (WSN) currently represent the best candidate to be adopted as the communication solution for the last mile connection in process control and monitoring applications in industrial environments. Most of these applications have stringent dependability (reliability and availability) requirements, as a system failure may result in economic losses, put people in danger or lead to environmental damages. Among the different type of faults that can lead to a system failure, permanent faults on network devices have a major impact. They can hamper communications over long periods of time and consequently disturb, or even disable, control algorithms. The lack of a structured approach enabling the evaluation of permanent faults, prevents system designers to optimize decisions that minimize these occurrences. In this work we propose a methodology based on an automatic generation of a fault tree to evaluate the reliability and availability of Wireless Sensor Networks, when permanent faults occur on network devices. The proposal supports any topology, different levels of redundancy, network reconfigurations, criticality of devices and arbitrary failure conditions. The proposed methodology is particularly suitable for the design and validation of Wireless Sensor Networks when trying to optimize its reliability and availability requirements.

## 1. Introduction

Traditionally, applications in industrial environments are based on wired communication solutions [1]. However, recently, the industry has shown interest in moving part of the communication infrastructure from a wired to a wireless environment, in order to reduce costs related with installation, maintenance and scalability of the applications. In this context, Wireless Sensor Networks (WSN) actually represent the best candidate to be adopted as the communication solution for the last mile connection in process monitoring and control applications in industrial environments [2]. Among many advantages, the absence of a wired infrastructure enables WSN to extract information in a simpler way than traditional monitoring and instrumentation techniques [3].

Industrial applications have usually stringent dependability (reliability and availability) requirements, since faults may lead to system failures which can result in economic losses, environmental damage or hurting people [4,5]. In this context, we can classify faults as transient or permanent [6]. Transient faults usually affect communication links between devices and are caused by noise or electromagnetic interferences. Permanent faults affect network devices and have their origin in hardware malfunctions. After a permanent fault a device is considered (permanently) failed, and to become operational again a repair activity is necessary. In this paper we focus on permanent faults that affect network devices leading to its failure (note: the failure modes of a failed element become the fault types for the elements interacting with it [6]). Permanent faults have, typically, a major impact on the system operation [7]. Their immediate consequence is that communications with the affected device are no longer possible. However, in worst case situations various network devices can become isolated, as when a network device that acts as a router fails. As a result, the control algorithm is disturbed which may lead to a system failure with serious consequences.

The use of a methodology to evaluate the dependability requirements of a WSN can anticipate decisions regarding the topology, criticality of the devices, levels of redundancy and network robustness, that can be used to take decisions during the system life-cycle, and particulary, on early planning and design phases. For example, depending on the topology, alternative paths to the sink can be created improving the overall reliability of the network. In the same way, if a sensitivity analysis is supported, critical devices can be identified and decisions about different redundancy approaches can be taken.

The main contribution of this paper is to propose a methodology to evaluate the reliability and availability of Wireless Sensor Networks in industrial environments that are subject to permanent faults on network devices. The approach is based on Fault Tree Analysis (FTA), which is a technique used to obtain the probability of occurrence of an undesired state or event [8]. In the addressed case, the undesired event is related to the failure of a specific device or group of devices. A device is considered to be faulty if it suffers a permanent failure or if there is not any route to sink that includes the device.

The proposal addresses several aspects, being very flexible and able to be easily adapted to different kinds of scenarios. When compared with the available approaches, the main advantages are:

- Support of all possible topologies: line, star, cluster and mesh;
- Network failure conditions can be specified in a very flexible way, ranging from a single device to groups of devices;
- Failure and repair processes can be characterized using different types of time distribution functions;
- Network devices can have redundant (internal) architectures;
- Topology reconfigurations due to device failures are considered (e.g., self-healing routing protocols);
- Different types of dependability measures can be obtained from the same model (e.g., reliability, availability, MTTF) as well as the criticality of the network devices (Birnbaum’s measure).

To complement the proposed approach we have also developed a software tool that automatically evaluates the reliability and availability of a WSN. The tool automatically generates a fault tree with the minimal set of events that leads to the network failure condition. After that, the fault tree is translated into a language understandable by the sharpe (Symbolic Hierarchical Automated Reliability and Performance Evaluator) tool [9], which is used to compute the desired dependability measures.

The remainder of this paper is organized as follows: Section 2 surveys some of the most relevant research works on reliability and availability evaluation for Wireless Sensor Networks. In Section 3, we give an overview about Wireless Sensor Networks with a special attention to wireless industrial networks standards, such as WirelessHART and ISA 100.11a. Next, in the Section 4, is held a brief introduction to Fault Tree Analysis (FTA) and basic concepts used in the proposal. Section 5 describes the proposed methodology for the reliability and availability evaluation of Wireless Sensor Networks. In Section 6 several scenarios are evaluated using network topologies commonly adopted in industrial applications. Finally, Section 7 concludes the paper and presents directions for future studies.

## 2. Related Works

The network reliability problem is a classical reliability analysis problem [10] that can be classified as: *k-terminal*, *2-terminal* or *all-terminal*. Suppose a network with *N* devices and a set of *K* devices (*K ⊂ N* and *|K| < |N|*). *K* is a set composed by a sink node and *|K| −* 1 field devices. Defining a sink device *s ∈ K*, the *k-terminal* problem is expressed as the probability that there is at least one path from *s* to each field device in *K*. The *2-terminal* problem is the case where *|K|* = 2, whereas the *all-terminal* problem is the case where *|K|* = *|N|*. These cases are known to be NP-hard problems, however several algorithms can be found for networks with limited size [11].

The network reliability problem has been widely studied for wired networks. For example, in [12] the author deals with the problem of measuring the reliability and availability of a wired network assuming hardware and software failures. The author gives an important insight about the state-space enumeration and the topology adaptation strategy when failures occur. The main difference between the reliability analysis of wired and wireless networks is related to the dynamics of the network. In a wireless networks, the dynamics of the network is greater since links fail more often and also due to the mobility of some of the devices. An early work about the reliability evaluation for a radio-broadcast network was conducted by [10]. In that work, the authors considered unreliable devices and reliable links and showed that the two-terminal reliability problem for radio broadcast networks is computationally difficult.

In [13], the authors analyzed the reliability and the expected maximum delay for a distributed sensor network. The network is assumed to be dense and organized into clusters. The reliability was measured as the probability that there was at least one path between the sink device and a sensor node within a cluster. The authors assumed unreliable devices and reliable links. It was proved that the problem was, in general, NP-hard. However for a topology up to 40 devices the problem is still tractable. In [14], the network reliability was evaluated for mobile ad-hoc networks based on the 2-terminal problem. The authors assumed unreliable devices and dynamic network connectivity. The proposed algorithm, although not finding the minimal cut set for the network, can be extended for the type of static networks typically found in industrial applications. In [15], the authors analyzed the influence of adding redundant devices, in what concerns the reliability and availability of multi-hop wireless networks. This work provides an interesting discussion about the reliability and availability of a WSN, particularly if it is considered that a router node can be a redundant device.

A tentative effort to create a methodology to evaluate the reliability of a WSN infrastructure was performed in [16]. The authors created a scheme based on reduced ordered binary decision diagrams (ROBDD) to model a cluster topology, where a reliability evaluation was also conducted. The authors do not considered multiple paths connecting a device to the sink. Thus, it is no longer possible to use self-healing routing protocols. Common-cause failures were considered, but the technique was focused in a single cluster. The methodology was applied only for a cluster topology with non-flexible failure conditions, and the criticality of devices was not determined. By introducing the concept of coverage-oriented reliability, the same authors extended the previous work [16] creating other mechanisms to evaluate the reliability of a WSN [17]. They assumed that the network fails if a specific point in the cluster is not covered by at least *K* devices. This give a more flexible way to configure failure conditions. However it is not possible to create two or more coverage subsets for the same cluster.

Another coverage-oriented reliability mechanism was proposed in [18]. The authors propose a framework to evaluate the reliability of a WSN based on coverage requirements. Given an area *A*, the network fails if there is no subset of fully operating nodes whose own generated traffic can reach the sink and the total area covered by this subset is greater than *A*. The authors used a 3-state node reliability model to represent random failures in the devices. This model has been shown to work better over the conventional 2-state (operate/fail), but it neither supports the inclusion of spare devices nor indicates the criticality of the devices. Finally, the inability to create several coverage areas makes it difficult to specify flexible failure conditions.

Another methodology for the reliability evaluation of a WSN was proposed in [19]. The authors propose a new topology control mechanism and they used a methodology for evaluating the reliability of the network operating with this mechanism. The basic idea is to represent the network as a graph and to measure the reliability based on the number of functional spanning trees. If there is at least one functional spanning tree, then the network is considered reliable. The proposal is simple and works very well for the analysis of the topology control mechanism. However it is not suitable to evaluate arbitrary WSN. It is not possible to use and validate physical redundancy, neither to compute the criticality of the devices. Flexible failure conditions are also very difficult to represent due the failure dependences for a spanning tree condition.

As an alternative to the aforementioned approaches, Fault Tree Analysis (FTA) techniques can be used to evaluate the reliability and availability of the network. The main advantage of FTA is related to the intuitive procedure used to describe events that lead to network failures. However, for complex topologies the construction of the fault tree is a time-consuming task demanding much effort. The usual solution is to adopt an approach that automatically generates the fault tree based on the network specification. In [20], the authors developed a modeling methodology for automatic generation of fault trees. The idea is to split a system in different components that are represented by function tables and state transition tables. These components are connected to each other in order to describe the behavior of the whole system. After the modeling phase, a trace-back algorithm is used to create the fault tree. In [21], an automatic generation mechanism for the fault tree was described within the context of an automation system. The basic idea is to model the system using a timed automata and then perform a model checking to verify which situations may lead to a system failure. After that, the results are summarized and the fault tree is generated. Another way to automatically generate the fault tree is to use digraphs (directed graphs) [22]. A digraph is composed by nodes and edges. Nodes represent component failure whereas edges represent relationships between nodes. In [23], the authors developed an automatic generator for fault tree based on digraphs. This work was an improvement upon the algorithm previously proposed in [22]. In both approaches they create a digraph to model the behavior of the system. All aforementioned works use dependency relations between system components to generate a fault tree.

Recently, an interesting contribution to the dependability evaluation of Wireless Sensor Networks was proposed in [24,25]. The main idea is to compute a new dependability parameter called *producibility*, that measures the probability of a sensor node is in a active state and it is able to communicate with the sink at time *t*. This new measure combines the reliability of a sensor node with their battery level. Network failure conditions are related with the existence of a minimum number of sensor nodes (*k-out-n*) able to send data to the sink. Metrics are computed using analytic techniques based on Continuous Time Markov Chains (CTMC) and reward functions. In [26] the same authors propose an alternative approach based on Non-Markovian Stochastic Petri Nets (NMSPN). This numerical based approach was selected to relax some of the assumptions related to the analytical technique. In the same work, they also propose to use Fault Trees to compute the network failure condition. Although these are interesting works, they are too much focused on energy consumption problems, which makes it difficult to extend the proposed methodology for generic scenarios. The same applies for the metrics. Moreover, network failure conditions are defined in a very restrictive way (*k-out-n* devices) which are not suitable for industrial scenarios, where it is important to identify the failed device and not only the number of failed devices.

It becomes clear from the previous discussion that these works only provide partial solutions for the problem. Since most of them are focused on specific scenarios, they are very restrictive with regard to the definition of network failure conditions, dependability metrics, topology, network reconfiguration and redundancy aspects, as well as applicability to industrial scenarios. The present work aims to remove most of these limitations by proposing a methodology that considers the most important aspects of the network operation through a flexible approach.

## 3. Wireless Sensor Networks

Wireless Sensor Networks are a pervasive technology that targets the connectivity between sensor nodes in multiple environments. Its infrastructure is usually composed of a large number of sensor nodes, with small physical size, which runs upon relatively inexpensive computational processes. Sensor nodes measure local environmental conditions and forward sensed values to a set of central points, referred as sink nodes, for appropriate processing. Sensor nodes can sense the environment, communicate with neighbor nodes, and perform basic computations on collected data. Installation flexibility and easy configuration enable better usability and maintenance than traditional communication technologies [1]. These characteristics allow the use of WSN over a wide range of useful applications [3,27–29].

Currently, WSN solutions are based on standardized or proprietary protocols. There are many different protocols for the upper layers, but the IEEE 802.15.4 [30] is a *de facto* standard for the lower layers. Recently the IEEE 802.15.5 standard [31] has been released to provide multi-hop mesh functions. Both standards are compatible while maintaining simplicity. On the other hand, Zigbee (2004) and Zigbee Pro (2007) were the first standards to implement the upper layers. Both standards do not have the support for channel hopping and are still not scalable enough to support large topologies [32]. Channel hopping is an important feature when industrial applications are considered, due to its robustness against external interferences and persistent multi-path fading. A new standard, IEEE 802.15.4e, is being developed to support additional industrial requirements and it is expected to be approved by the end of 2011. Currently, only the WirelessHART and ISA 100.11a standards are suitable to be used in industrial applications.

The methodology proposed in this paper can be easily implemented to evaluate the reliability and availability of Zigbee, WirelessHART and ISA 100.11a networks. However, as the application focus is for industrial environments, only the WirelessHart and ISA 100.11a standards will be described in the following sections.

### 3.1. WirelessHART

WirelessHART is an extension of the HART protocol to support wireless communication. The concept behind WirelessHART was first discussed in 2004 at the HART Communication Foundation (HCF) meeting. The main question was how to interoperate legacy devices with wireless devices, in order to take advantage of the amount of installed HART devices. It is estimated that more than 24 million HART devices are installed around the world and its shipping expected are around over 2 million per year [33]. In September 2008, the WirelessHART specification (HART 7.1) was approved by the International Electrotechnical Comission (IEC) as a publicly available specification (IEC 62591) [34]. WirelessHART was the first industrial wireless communication technology to attain this level of international recognition [35].

**Figure 1.**
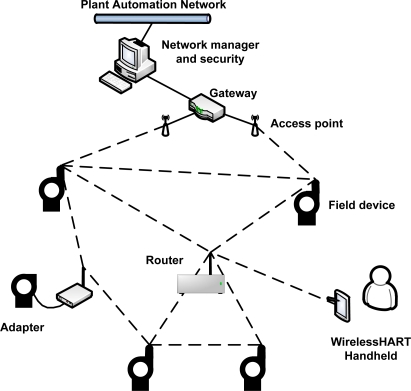
WirelessHART devices.

WirelessHART defines eight types of devices, as presented in Figure 1: network manager, network security, gateway, access point, field device, adapter, router and handheld device. All devices that are connected to the wireless network implement basic mechanisms to support network formation, maintenance, routing, security and reliability.

Field devices are the most basic WirelessHART devices. They are directly connected to the process and plant equipments. Field devices can transmit measurement data, receive and forward packets from/to any device. Usually they may be line, loop or battery powered. All field devices have a physical maintenance port, which is used for offline configuration and diagnostics. Compatibility with legacy HART devices is guaranteed through the use of adapter devices. The adapter devices are not directly connected to the plant equipments, however, they have to support the same functionalities of field devices. On the other hand, handheld devices are used during the installation, configuration and maintenance phases of the network. They do not have to support routing mechanisms.

Router devices are used for routing purposes, *i.e.*, forward packets from one device to another device. They are not directly connected to the industrial process, thus they can be installed anywhere in the plant. Their use is not really necessary since field devices have internal routing capabilities. However, router devices can provide redundant paths to the gateway, and they can also minimize energy consumption in field devices. The connection between the plant automation network and the wireless network is provided by the gateway. The gateway works as a sink point for all wireless traffic. The logical communication with the wireless network occurs through access points installed in the gateway. The amount of access points can be configured to increase redundancy and to improve the effective network throughput.

The security manager is the entity responsible for ensuring the security over the network. It provides join, network and session keys for all devices. These keys are used to authenticate and to encrypt data. The storage and management of keys is also under the responsibility of the security manager. The core of the WirelessHART is the network manager. It is logically connected to the gateway and manages the entire network. The communication with network devices occurs through the application layer protocol. The main duties of the network manager are related with scheduling, management of the device list, routing (redundant paths), collect information about performance, failure detection, and network formation.

WirelessHART has a physical layer based on IEEE 802.15.4, but implements its own medium access control (MAC) sublayer. The MAC is based on a TDMA (Time Division Multiple Access) communication mechanism that uses *superframes*. Superframes are composed by slots, and the amount of slots indicates its periodicity. To support multiple schedule requirements, a WirelessHART network can use multiple superframes with different number of slots. Each slot has a fixed duration of 10 ms, which is enough time to transmit a packet and receive an acknowledgment (the maximum packet size is 133 bytes including headers). Slots can be dedicated or shared. The use of dedicated slots is more common. Shared slots are used for transmission retries and advertising indication during the join procedure. A slot supports until 15 channels, thus, theoretically 15 devices can simultaneously transmit in the same slot time. The standard uses a mechanism of frequency hopping and a channel blacklist to minimize the influence of noise/interference in the network operation and consequently to increase the communication reliability.

An important procedure defined in the WirelessHART is the path failure indication [36]. The communication between two devices can fail due to hardware failures or due to interferences from the external environment. Therefore, it is essential that failure events are reported to the application. The WirelessHART defines the variable *path-fail-time* to control the path failure indication. If a device identifies that no packet was received from a specific neighbor within the *path-fail-time*, an alarm indicating that the path is no longer available is sent to the application.

### 3.2. ISA 100.11a

The International Society of Automation (ISA) has developed a wireless mesh networking standard known as ISA 100.11a [37] that guarantees a deterministic communication latency, while increasing the communication reliability. It focuses on process control and monitoring applications, with latency requirements around 100 ms. ISA 100.11a can coexists with other wireless technologies such as cell phones, IEEE 802.11*×*, IEEE 802.15*×*, IEEE 802.16*×*, and can provide tunneling for legacy protocols (HART, Foundation Fieldbus, Profibus, Modbus).

**Figure 2.**
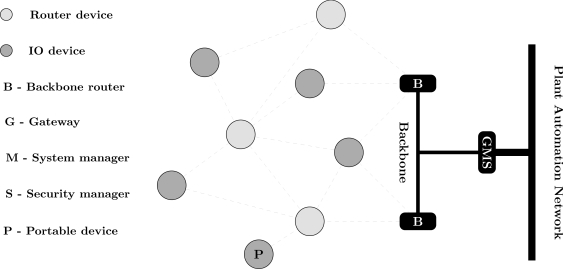
Typical ISA 100.11a network.

A typical ISA 100.11a network is presented in Figure 2. It may be composed of seven types of devices: gateway, system manager, security manager, router, backbone router, input/output (IO) devices and portable devices. Each device has a specific role definition that control its functions. The IO device is responsible for monitoring the environment. If minimization of the energy consumption is configured, the IO device only transmits messages. Otherwise, the IO device can also route messages. In addition to the routing functionality, a router device shares the function of provisioning devices to join the network. A router device can use slow slotted hopping to send advertising messages about the network for joining devices. On the other hand, backbone router devices are used to encapsulate external networks in order to carry native protocols over ISA 100.11a. The gateway device provides a connection between the wireless sensor network and the plant automation network. There is support for multiple gateways and backbone routers [38]. The most important tasks are performed by the security manager and the system manager. The system security management function is controlled by the security manager whereas the system manager governs all the network, devices and communications.

Similarly to WirelessHART, ISA 100.11a has a physical layer based on IEEE 802.15.4. On the other hand, the data link layer is slightly different from the one used on WirelessHART. The slot time has duration of 10 ms or 12 ms. The schedule mechanism was designed in a more flexible way than the WirelessHART schedule. There is support for slotted channel hopping (TDMA), slow channel hopping (CSMA) and a hybrid combinations of both. The TDMA approach is similar to the WirelessHART schedule. In the CSMA (Carrier Sense Multiple Access) approach, contiguous slot times are grouped into a single radio channel with a period ranging from 100 ms to 400 ms. During this period the radio of devices are always activated. This approach is indicated for neighborhood discovery procedures and frequency hopping in the case of overlapping with 802.11*×* networks, for example. On the other hand, the hybrid approach is more suitable for a flexible retry procedure. Other improvements when compared with the WirelessHART standard are related with the frequency hopping pattern. ISA 100.11a has defined five default hopping patterns to mitigate the influence of external communication interference. For example, the pattern 1 is configured to eliminate the overlap with the same channels of IEEE 802.11*×*.

The network and transport layers support mesh networks, similarly to WirelessHART. However, addressing in ISA 100.11a is compatible with the 6LoWPAN [39] (IPv6 over IEEE 802.15.4). ISA 100.11a also introduces a new mechanism to detect failures in the network based on the transmission of alert messages.

## 4. Reliability, Availability and Fault Tree Analysis

In this section, we provide a brief introduction to reliability, availability and fault tree analysis concepts that are closely related with the proposed methodology.

### 4.1. Reliability

Reliability is a measure used to characterize if a component/system, is properly working according to its specifications during a specific period of the time [6]. Formally, it is defined as the probability that a component does not fail in the time interval (0*, t*]. Considering that the *time to failure* of a component, *T*, is a random variable defined by a cumulative distribution function *F* (*t*) (CDF), the reliability *R*(*t*) is given by:

(1) R(t)=Pr(T>t)=1−F(t)

The reliability function is closely related with the *failure rate function* λ(*t*). This function (also known as *hazard rate*) describes the instantaneous failure rate of a component. Formally, this function is defined as the probability that a component fails during the period of the time [*x, x* + Δ*t*], knowing that it is working at time instant *t* = *x*. The behavior of this function has been extensively discussed in the literature [40]. For many systems/components this function presents a characteristic shape which is similar to a *bathtub curve*. When the system is young, the failure rate is higher (infant mortality), and then quickly decreases until stabilizes (useful life). As the system/component gets older it increases again (wear out). For electrical/electronic systems it is common to consider that the failure rate is constant during the useful life period, *i.e.*, λ(*t*) = λ [41]. It can be proved that *R*(*t*) and λ(*t*) are related according to the following expression [42]

(2) $$R(t)=\exp\left(-\int_{0}^{t}\lambda (u)\mathrm{du}\right)$$

Therefore λ(*t*) establishes *R*(*t*). Another metric related with *R*(*t*) is the MTTF (*Mean Time to Failure*). Formally, it is defined as the expected (average) time during which a component is working properly, and is given by [42]

(3) $$\mathrm{MTTF}=E(T)=\int_{0}^{\infty }\mathrm{tf}(t)\mathrm{dt}$$

### 4.2. Availability

Availability is a measure which is defined as the probability of a component/system is functioning at time *t*. The availability at the instant *t* is referred as *instantaneous availability* *A* (*t*). The *steady-state availability* expresses the percentage of time that a component is working properly. Formally it is defined as *A* = lim*_t_*_→∞_ *A*(*t*) (note: this metric only makes sense in systems which have a stationary probabilistic condition [42]). Availability is closely related with repair actions. In fact, it is implied that the system is repaired after a failure, otherwise lim*_t_*_→∞_ *A*(*t*) = 0. For a non-repairable system *A*(*t*) = *R*(*t*).

Similarity to the failure rate λ(*t*), it is possible to define a *repair rate* *μ*(*t*), as the rate at which a failed component is repaired. The MTTR (*Mean Time to Repair*) is defined as the expected (average) time that takes to repair a component. If failure and repair rates are assumed constant, respectively λ and *μ*, then it can be proved that *A*(*t*) is given by [42]

(4) $$A(t)=\frac{\mu }{\lambda +\mu }+\frac{\lambda }{\lambda +\mu }e^{-(\lambda +\mu )t}$$

Finally, if failure and repair actions are independent and described by *i.i.d* (independent and identically distributed) random variables, than the following relationship applies [42]

(5) $$\underset{t\to \infty }{\text{lim}}A(t)=A=\frac{\mathrm{MTTF}}{\mathrm{MTTF}+\mathrm{MTTR}}$$

This expression is independent of the CDF that characterize failure and repair processes.

### 4.3. Fault Tree Analysis

Fault tree analysis (FTA) is a deductive technique commonly used to evaluate system’s dependability [43]. It can be used to describe the root causes that lead to a system failure, in a qualitative or quantitative way. In the former case, it can be used during system development to identify potential problems that could lead to a system failure, or after commissioning, to identify events that caused a system failure. In the latter case, it is mainly used to obtain dependability measures, such as the system’s reliability and availability.

**Figure 3.**
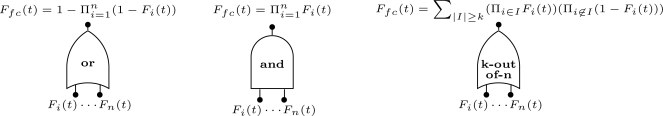
Cumulative distribution function (*F_fc_*(*t*)) for the gate output (*and*, *or*, *k-out-of-n*).

Fault trees (FT) are a graphical model that represents the combination of events that lead to a system failure. The model uses a treelike structure composed by events and logic gates. Events represent either normal or faulty conditions, such as component failures, environmental conditions, human-made faults, *etc*. They are considered *boolean*, *i.e.*, they either occur or not occur. Logic gates are used to represent the cause-effect relationships among events. The inputs of these gates are either single events or combinations of events which result from the output of other gates. There are several types of gates available, such as *and*, *or* and *k-out-of-n* (Figure 3). The process of building a FT is performed deductively and starts by defining the top *event*, which represents the *system failure condition*. From this event, and by proceeding backwards, the possible root causes are identified. The events at the bottom of the tree are referred as *basic events*. If a basic event occurs two or more times in a FT it is called a *repeated event*.

From a probabilistic point of view, the assessment of a FT consists of calculating the probability of the top event starting from the probabilities of the basic events. This calculation is performed differently for each type of gate. Assuming a gate with *n* independent inputs (events), where the occurrence of event *i* is described by means of a cumulative distribution function *F_i_*(*t*), then the gate output CDF *F_fc_*(*t*) is given according to the Figure 3 [42].

When an *and* gate is used, the failure condition occurs only if all input events have occurred. On the other hand, when an *or* gate is used, the failure condition occurs if at least one input event have occurred. Finally, if a *k-out-of-n* gate is used, the failure condition occurs if at least *k* input events have occurred.

When a FT does not contain any repeated event, the probability of the top event can be obtained through direct calculation using the formulas presented in Figure 3. However, if there are repeated events these equations are no longer valid. Therefore, in these situations it is necessary to employ different approaches. In the literature we can find several techniques to accomplish this task, such as inclusion-exclusion principle, sum of disjoint products, factorization and direct/indirect recursive methods [43]. In the context of this work, we will focus on the *sum of disjoint products* (SDP) [44].

The SDP method can be efficiently employed in fault trees with repeated events and it is easily automated. The basic idea of SDP is to find a boolean function *ϕ*(x) that describes the failure condition (*i.e.*, the top event), and to transform this function into another function where the individual terms are mutually exclusive.

Consider a system with *n* components. The first step starts by obtaining the *structure function* of the system, *ϕ*(x), which is given by

(6) $$\phi (x)=\{\begin{array}{ll} 1 & \text{if the system has failed}\\ 0 & \text{if the system has not failed} \end{array}$$

where **x** is referred as the *state vector*, x = (*x*_1_*, x*_2_, . . ., *x_n_*). Each element *x_i_* is a boolean variable that represents the state of component *i* (e.g., *i* = 1 ⇔ the component has failed). The function *ϕ*(x) can also be expressed as the union of *minimal cut sets*.

(7) $$\phi (x)=K_{1}\cup K_{2}\cup \cdots \cup K_{n}$$

A cut set *K_i_* is a subset of events whose simultaneous occurrence leads to the occurrence of the top event. A cut set is said to be *minimal* if does not contain other cut set. There are several algorithms available to automate the process of obtaining the minimal cut sets from a fault tree [43]. After obtaining the cut sets, *ϕ*(x) can be transformed in a sum of disjoint products, as follows

(8) $$\phi (x)=K_{1}\cup K_{2}\cup \cdots \cup K_{n}=K_{1}\cup {\bar{K}}_{1}K_{2}\cup \cdots \cup {\bar{K}}_{1}\cdots {\bar{K}}_{n-1}K_{n}$$

where *K_i_* the *i*-cutset and *K̄_i_* its complement. Since the terms are pairwise disjoint, the probability of the top event can be obtained as the sum of the probabilities of the individual terms.

It is possible to compute several dependability measures from a fault tree. In the context of this work we will focus on reliability and availability. Assume that the top event represents the failure of the system. Thus, the probability of this event occurring during a period of time *t* is the complement of the reliability *R*(*t*). If the top event is expressed by its *minimal cut sets*, then to compute the reliability is only necessary to replace each event *i*, in the respective cut set, by its reliability function *R_i_*(*t*). After that, the reliability of the system *R*(*t*) can be easily computed using simple probability laws (*i.e.*, probability of union and intersection of events).

Availability can be obtained in a similar way, by replacing each event by the availability function of each component *A_i_*(*t*). However, this computation in only valid if the repair processes are all independent and if the number of repairman (*i.e.*, number of repair actions) is not limited. Further details can be found in [43].

### 4.4. Component Importance

After computing the top event probability (or any other relevant metric, such as the reliability or availability), the user is able to foresee the system behavior from a dependability viewpoint. However, this does not highlight what is the contribution of each component to the final result. Such information is relevant because it allows the system designer to make decisions concerning the system structure, which can be used to optimize dependability metrics (e.g., availability), or other performance measures.

In this section we will review some importance measures that can be used to rank components in order of importance. We assume a system composed of *n* independent components, where each component *i* is characterized by a reliability function *R_i_*(*t*).

#### 4.4.1. Birnbaum’s Measure

Birnbaum’s measure *I^B^*(*i|t*) is a metric that describes the reliability importance of a component [45]. This measure is defined as the partial differentiation of the system reliability with respect to the reliability of component *i*, as follows

(9) $$I^{B}(i|t)=\frac{\partial R(t)}{{\partial R}_{i}(t)}\;\;\text{for}\;\;i=1,2,\ldots ,n$$

If *I^B^*(*i|t*) is large, a small variation in the reliability of component *i* will result in a major change in the reliability of the system. A component *i* is considered *critical* for the system, if when the component *i* fails, the system also fails. Thus, the Birnbaum’s measure can also be interpreted as the probability of component *i* being critical for the system at time *t* [42].

#### 4.4.2. Criticality Importance

The criticality importance *I^CR^*(*i|t*) is a measure particularly suitable for prioritizing maintenance actions [42]. This measure is defined as the probability that component *i* is critical at time *t* and is failed at time *t*, knowing that the system is failed at time *t*, being defined as follows

(10) $$I^{\mathrm{CR}}(i|t)=\frac{I^{B}(i|t)\left(1-R_{i}(t)\right)}{1-R(t)}$$

In other words, the criticality importance is the probability that a component *i* has caused a system failure, knowing that system is failed at time *t*.

## 5. Methodology for Reliability and Availability Evaluation

The main objective of the proposed methodology is to provide a framework to support the evaluation of the dependability of a WSN, in order to provide valuable information to the system designer enabling it to develop robust and fault tolerant applications. The methodology can be applied on all stages of the network life cycle, allowing the identification of weaknesses (e.g., topology, devices, *etc*.) as well as helping to define a strategy to cope with these problems.

### 5.1. Introduction

As aforementioned in the Section 2, the reliability evaluation of a general network is a NP-hard problem. Nevertheless, as it will be discussed in the next section, this problem can be tractable for a low-medium number of field devices, as is the case of networks typically found in industrial applications.

**Figure 4.**
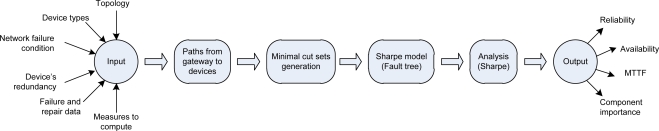
Overview of the methodology for reliability and availability evaluation.

Figure 4 overviews the proposed methodology. The process starts by providing information about the network topology, device types and redundancy, device’s failure and repair process and network failure condition. The latter one is defined by a logical expression that combines the failure status of field devices. For attaining flexible failure conditions and to support self-healing routing protocols, it is necessary to find all paths between the gateway (sink) and the devices that encompass the failure condition. Next, a fault tree is generated using all the previous data. From that, the respective minimal cut sets are obtained using an inversion technique. This cut set is re-expressed as a (minimal) fault tree, which is used to produce input data for the tool that computes the results. For this task we use the sharpe tool [9], which is able to compute the metrics of interest, either symbolically or numerically. It is possible to evaluate the reliability, availability and mean time to failure (MTTF) of the WSN, and also the Birnbaum’s and the criticality measures for all field devices. Finally, we also have developed a software tool that automates the previous steps.

### 5.2. Assumptions

The main assumptions considered in the methodology are the following:

- **Topology:** the network is composed of *N* field devices, which can belong to one of the following types: end device (e.g., sensor/actuator node), router, access point and gateway (*i.e.*, sink). Devices are arranged according to one of the following topologies: line, star, cluster and mesh. These elements are defined according to the WirelessHART, ISA 100.11a and Zigbee standards;
- **Faults:** only permanent faults are considered. The links, due to its wireless nature, are only affected by transient faults and thus are considered to be reliable (*i.e.*, they do not fail). Thus, only field devices can fail. After a permanent fault a device is considered failed (permanently). We assume that device failures are independent. In principle any type of distribution can be used to characterize the occurrence of device failures. However, the sharpe tool poses some restrictions. The tool imposes that CDFs must be expressed using *exponential polynomial* terms as following
  (11) $$F(t)=\sum_{j=1}^{n}a_{j}\;t^{k_{j}}\;e^{b_{j}\;t}$$
  Many distributions can be expressed in this way (e.g., exponential, Erlang, hypoexponential, hyperexponential). Other distributions (e.g., Weibull, deterministic) can be approximated using exponential polynomial terms. Further details can be found in [46].
- **Repairs:** field devices can be repaired after failing, if necessary. After a repair the device is considered as new. We consider that repair processes are independent and that the number of repairman (*i.e.*, number of repair actions) is not limited. The time necessary to repair a device is characterized by a *repair distribution*. This distribution is defined in analogous way to the failure distribution discussed previously;
- **Redundancy:** field devices can have an internal redundant architecture with several available spares. We assume that when the main element fails, its replacement by a spare is always performed with success;
- **Reconfiguration:** when a device fails the network topology can change. We assume that the network manager (WirelessHART) or system manager (ISA 100.11a) is able to identify with success a device failure, and then update the network topology (communication paths). It is also assumed that the time required to perform this operation is negligible and that it is always successful (if alternative paths exist). Thus, the support of self-healing routing protocols is assured, since all paths between a field device and the gateway are considered;
- **Measures:** the following measures can be computed: reliability, unreliability, availability, unavailability, MTTF and component importance (Birnbaum and Criticality). Results can be presented both numerically and symbolically using exponential polynomial terms.
- **Inputs:** to compute the measures it is necessary to provide the following input data: network topology, type of devices, device’s redundancy, network failure condition, characteristics of the device’s failure and repair processes and measures to compute.

### 5.3. Topology

**Figure 5.**
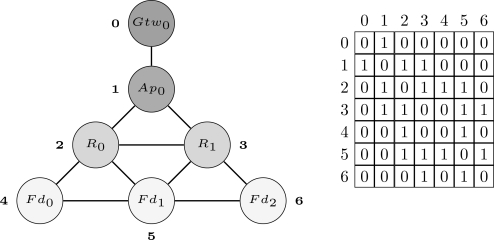
Example of a Wireless Sensor Network represented through a graph and its respective adjacency matrix.

The first step of the proposed methodology is to define a structure through which the network can be modeled. In the proposed approach, the network is organized as a graph *G*(*V, E*) with *n* vertices (*V*) and *k* edges (*E*). The vertices represent field devices whereas the edges represent the wireless links between devices. The network topology can be stored in the adjacency matrix (*A_n×n_*) of graph *G*. If a device *N_i_* has a neighbor *N_j_*, then the entries *a_ij_* and *a_ji_* of *A* will receive the value 1, otherwise they will receive the value 0. Thus, by using this structure we can represent any WSN topology. Figure 5 shows an example of a WSN represented using the aforementioned structure. In this example, the network is composed by 1 gateway (*Gtw*_0_), 1 access point (*Ap*_0_), 2 routers (*R*_0_ and *R*_1_) and 3 field devices (*Fd*_0_, *Fd*_1_ and *Fd*_2_). The indexes located more close to the vertices are used to identify the devices in the adjacency matrix.

### 5.4. Network Failure Condition

**Figure 6.**
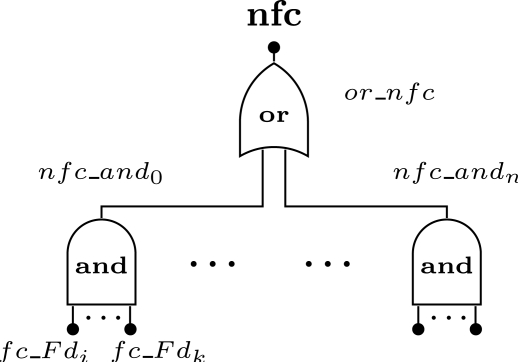
Network failure condition (nfc).

The network failure condition defines which combination of devices may lead to a network failure. In the proposed methodology we support any combination that can be expressed using boolean operators (*i.e.*, *AND*, *OR*). The failure condition associated to field device *N_i_* is defined as *fc_Fd_i_*. The case where the device failure condition is related with several failure events will be described in the next sections. A combination of devices that lead to a network failure is defined as *nfc_and_j_*, where *j* is the identification of combination and is represented by the boolean *AND* of the failure condition of the devices (note: if the *AND* gate has only one input, the event *nfc_and_j_* is replaced by the respective input). A device can belong to more than one failure condition. The network failure condition (*nfc*) is represented by the boolean *OR* of all combinations that lead to the network failure (Figure 6).

### 5.5. Device Redundancy

Regarding to redundancy issues, we consider that there are two types of field devices: with redundancy and without redundancy. The latter are simple field devices, while the former are composed by multiple devices arranged according to a fault-tolerant architecture based on a *hot standby sparing* approach. Hot standby sparing provides redundancy through the use of spare devices. A device is kept operational whereas the others devices (spares) are in standby. When the operational device fails, a spare module assumes the operation. We assume that this is an internal arrangement of the field device. That is, from the perspective of an external observer the behavior of a redundant device is indistinguishable from a device without redundancy. The number of spare devices available for each field device is an input of the model.

In the proposed model, devices are represented based on the failure event. The failure event for a redundant device is represented by the boolean *AND* of all spare devices whereas a device without redundancy is represented by a basic failure event.

### 5.6. Device Failure

After obtaining an expression for the network failure condition, it is necessary to define the conditions that may lead to the failure of a field device. Note that only devices that belong to the network failure condition are analyzed. We consider two possibilities for a device failure: (i) its hardware has failed; (ii) there is no path between the device and the gateway. This latter case corresponds to a *connectivity failure*, since the device itself did not failed in a strict sense (*i.e.*, it works), but it is considered non-operational from a network perspective because it is no longer possible to communicate with it. If a device along the path fails, the network may have the required mechanisms to reconfigure itself in order to use other paths. This type of reconfiguration is done by self-healing routing protocols. As aforementioned, the failure condition for a field device, *fc_Fd_i_*, is split in two involving hardware and connectivity problems.

**Figure 7.**
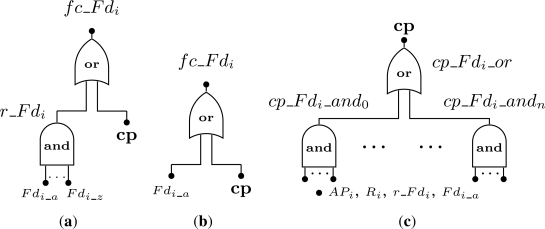
Device failure condition. **(a)** Redundant field device; **(b)** Simple field device; **(c)** Connectivity problem.

Regarding to hardware failures, we must consider the cases where the field devices are configured with or without redundancy. A redundant device fails (Figure 7(a)) if the current operating device suffers an hardware failure (*Fd_i_a_*) and if all its spares have already failed (events *Fd_i_b_* to *Fd_i_z_*). This is represented by event *r_Fd_i_*. On the other hand, for a device without redundancy (Figure 7(b)) the device fails when its hardware fails.

Regarding failures related with the connectivity problem (represented by the event *cp* in Figure 7(a,b), a device is considered to be faulty if there is no path from the device to the gateway (*i.e.*, sink). In other words, if a device has *j* paths connecting it to the gateway, at least one path must be working properly to consider that the device is operational. The event *cp* that represents this situation (Figure 7(c)) results from the combination of failures in access points (*AP_i_*), routers (*R_i_*), redundant devices (*r_Fd_i_*) and devices without redundancy (*Fd_i_a_*).

**Figure 8.**
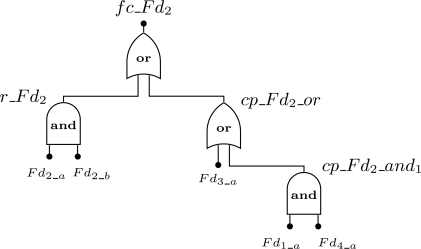
Example of a failure condition defined for the field device *Fd*_2_.

We will use an example to clarify the notation described in Figure 7. Consider a WSN composed by 4 field devices: *Fd*_1_, *Fd*_2_, *Fd*_3_ and *Fd*_4_. The addressed problem is to find the failure condition associated to device *Fd*_2_. Assume that device *Fd*_2_ is redundant and has one spare device, while devices *Fd*_1_, *Fd*_3_, and *Fd*_4_ are not redundant. Regarding to the connectivity problem, if device *Fd*_3_ fails or if devices *Fd*_1_ and *Fd*_4_ fail, then device *Fd*_2_ will also fail since there is no path to the gateway. Based on this scenario, the failure condition for device *Fd*_2_ (*fc_Fd*_2_) is presented by Figure 8. Note that the event *cp_Fd*_2_*_and*_0_ was replaced by a single event *Fd*_3_*a*_ because it makes no sense to build an *AND* gate with just one input.

**Algorithm 1:**
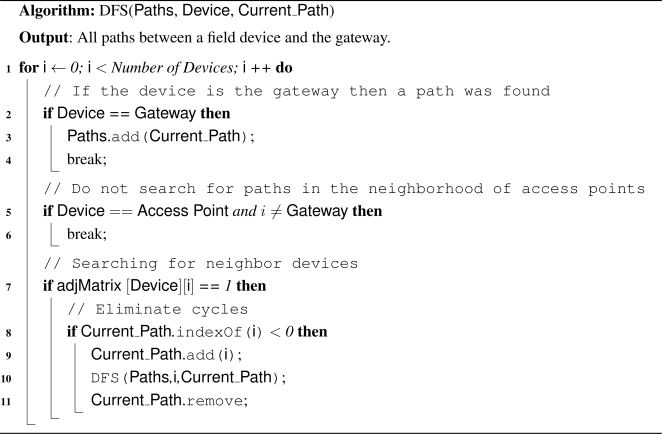
Algorithm to generate all paths between a device and the gateway.

Note that to find all combinations that lead to a connectivity failure necessarily requires some effort. To attain this, it is necessary to search all paths between the gateway and field devices that belong to the network failure condition. This procedure is described in Algorithm 1. The basic idea is to perform a depth-first search (DFS) in the adjacency matrix that represent the WSN. The procedure recursively traverse all devices on the path until the gateway is reached. Two restrictions were introduced to simplify the DFS. First, it makes no sense to search neighbor devices of the access point (line 5). Access points are directly connected to the gateway, and an access point does not communicate with another access point. The second restriction is related to the elimination of paths within a cycle. During the recursion, a device only joins to the current path if it is not already part of that path (line 8).

**Figure 9.**
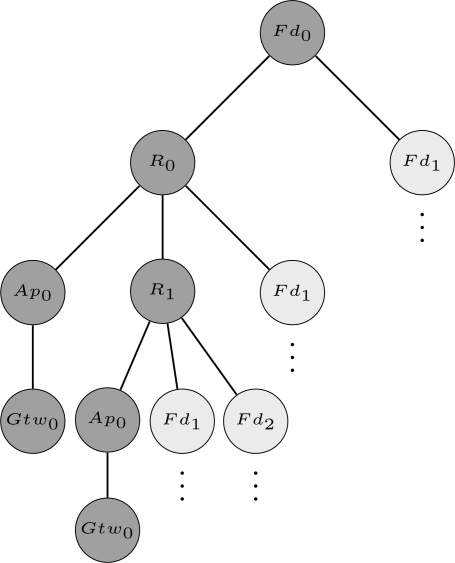
Output flow of Algorithm 1 for the device *Fd*_0_ of Figure 5.

For the sake of understanding Algorithm 1, considers the example of the output flow produced when the paths from device *Fd*_0_ to the gateway are being searched for the scenario presented in Figure 5. The output flow for this example is shown in Figure 9 (note: only two paths are described (dark gray circles) due to lack of space, however the others paths can be easily deduced.). The output flow starts at device *Fd*_0_ and explores as far as possible each branch (neighbor) until reaching the gateway. After that, a backtracking procedure is conducted and another branch will be evaluated. Figure 9 is composed by 5 levels. The first level is composed only by the target device (*Fd*_0_). The second level is composed by the neighbors of device *Fd*_0_ (*R*_0_ and *Fd*_1_). The other levels follow the same procedure taking into account the restrictions imposed by Algorithm 1 (lines 5 and 8).

All paths generated by Algorithm 1 are stored in a data structure based on a fault tree. This data structure will be used to generate the minimum cut sets in the next section. Each path *i* belonging to device *j* is mapped for an *and* gate. The inputs of each *and* gate are composed by the devices of the respective path. An *or* gate is used to connect all the paths (*and* gates) from a device to the gateway.

### 5.7. Minimal Cut Set Generation

After defining both the network failure condition and finding all the paths between the gateway and field devices that belong to the network failure condition, it becomes possible to generate a fault tree describing the network failure process. In this faulty tree, the top event represents the network failure condition and the basic events represent the device failure events. It could have been possible to feed this model directly into an evaluation tool in order to compute the dependability measures. However we choose to reduce this tree to a *minimal* one. The goal is to obtain a less complex fault tree in order to enable a faster computation of the dependability measures. Therefore, we compute the minimal cut sets of the fault tree and use this data to build a *minimal* fault tree.

The algorithm used to compute the minimal cut sets is similar to the one proposed in [47]. The major difference is that in the original proposal the authors consider reliable devices and unreliable links, whereas in our proposal we consider the opposite (*i.e.*, unreliable devices and reliable links). An inversion technique to generate the minimal cut sets from the *minimal path sets* (MPS) was proposed in [48]. A MPS represents a set of components such that, if all components are properly working then the system is operational. However, if a component of the MPS fails, then the system also fails. It was proved in [48] that the application of DeMorgan’s law to an appropriate boolean polynomial of the minimal path set is related to a boolean polynomial of the minimal cut set. An efficient algorithm for path inversion was extended in [47] based on the procedure proposed in [48].

**Figure 10.**
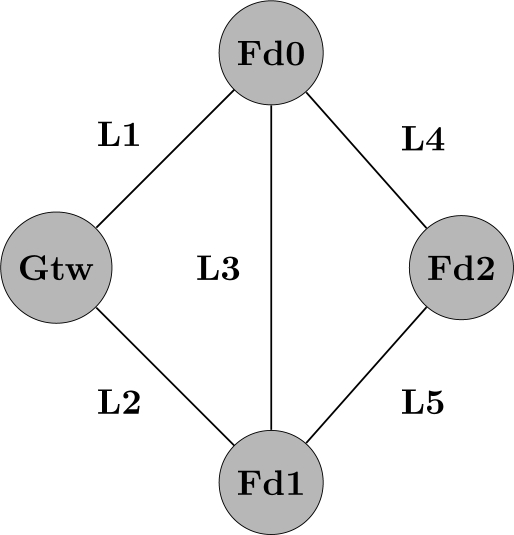
Topology used to exemplify the minimal cut set generation.

To clarify the minimal cut set generation, consider the following example that assumes the topology described in Figure 10. In this scenario, the field device *Fd*_2_ is the source whereas the gateway is the sink. *L_i_* indicates if a link between two network devices is operational, whereas $\bar{L_{i}}$ indicates the link is down. Based on [47], the minimal path set for this example is described by Equation (12).

(12) $$L_{1}L_{4}+L_{2}L_{5}+L_{2}L_{3}L_{4}+L_{1}L_{3}L_{5}$$

As described in [48], applying the DeMorgan’s laws to the Equation (12) we can find a boolean polynomial related to the minimal cut set according to Equation (13). By applying the distributive law ((*A* + *B*)*C* = *AC* + *BC*) and the absorption law (*A* + *AB* = *A*) to Equation (13), we obtain the result presented in Equation (14). In other words, the communication between *Fd*_2_ and the gateway is broken if either the links *L*_1_ and *L*_2_ fail or if the links *L*_1_, *L*_3_ and *L*_5_ fail or if the links *L*_2_, *L*_3_ and *L*_4_ fail or if the links *L*_4_ and *L*_5_ fail.

(13) $$\left(\bar{L_{1}}+\bar{L_{4}}\right)\left(\bar{L_{2}}+\bar{L_{5}}\right)\left(\bar{L_{2}}+\bar{L_{3}}+\bar{L_{4}}\right)\left(\bar{L_{1}}+\bar{L_{3}}+\bar{L_{5}}\right)$$

(14) $$\bar{L_{1}L_{2}}+\bar{L_{1}L_{3}L_{5}}+\bar{L_{2}L_{3}L_{4}}+\bar{L_{4}L_{5}}$$

On the other hand, if we assume unreliable devices and reliable links the minimal path sets of Figure 10 would be different from the represented in Equation (13). In this case, the minimal path sets are described by Equation (15). In the same way, if we apply the DeMorgan’s law and use the distributive and the absorption laws, the minimal cut sets for the example of Figure 10 can be obtained according to Equation (16). In other words, the communication between *Fd*_2_ and the gateway is interrupted if either the gateway fails or if *Fd*_2_ fails or if *Fd*_0_ and *Fd*_1_ fail.

(15) $${\mathrm{Fd}}_{2}{\mathrm{Fd}}_{0}\mathrm{Gtw}+{\mathrm{Fd}}_{2}{\mathrm{Fd}}_{1}\mathrm{Gtw}+{\mathrm{Fd}}_{2}{\mathrm{Fd}}_{0}{\mathrm{Fd}}_{1}\mathrm{Gtw}+{\mathrm{Fd}}_{2}{\mathrm{Fd}}_{1}{\mathrm{Fd}}_{0}\mathrm{Gtw}$$

(16) $$\bar{\mathrm{Gtw}}+\bar{{\mathrm{Fd}}_{2}}+\bar{{\mathrm{Fd}}_{0}}\bar{{\mathrm{Fd}}_{1}}$$

### 5.8. Sharpe Source Code Generation

After computing the minimal cut sets it becomes possible to generate a *minimal* fault tree that represents the network failure process. As discussed previously, we choose to proceed in this way to enable faster computations, but also due to the fact that the sharpe tool does not accept models expressed as cut sets, but accept models described as fault trees.

**Figure 11.**
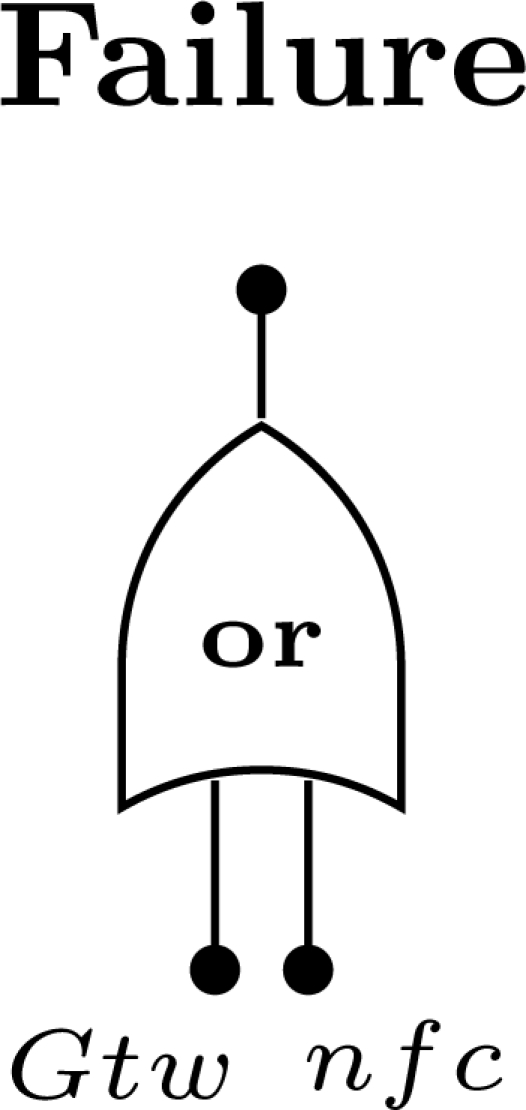
Events that conduct to a network failure.

In this *minimal* fault tree the top event results from two events. The first one is related to the failure of the gateway, whereas the second one is related to the network failure condition that is application dependent. The top events are described in Figure 11. Note that event *nfc* is based on the Figures 6 and 7 (Sections 5.4 and 5.6).

**Figure 12.**
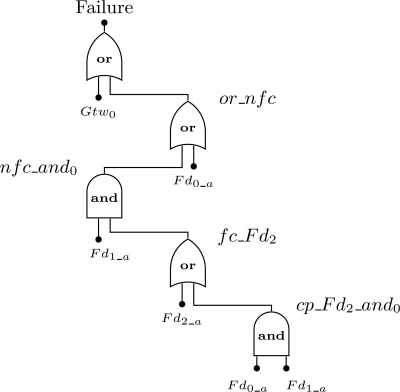
top event definition for the example of Figure 10, when the network failure condition is configured for *Fd*_0_ + *Fd*_1_ · *Fd*_2_.

For the sake of understanding of the top event definition, consider the example represented in Figure 10. We assume that the failure condition is defined as the following: *Fd*_0_ + *Fd*_1_ *· Fd*_2_. In other words, the network fails if the device *Fd*_0_ fails or if devices *Fd*_1_ and *Fd*_2_ fail. We also assume that all devices are configured without redundancy. The first step is to find the device failure condition (*fc_Fd_i_*) for each device present in the network failure condition. In this case, *fc_Fd*_0_ = *Fd*_0_*a*_, *fc_Fd*_1_ = *Fd*_1_*a*_ and *fc_Fd*_2_ = *Fd*_2_*a*_ + *Fd*_0_*a*_ · Fd_1_*a*_. The top event for this example is presented in Figure 12.

The following step is to transform the generated fault tree into an input file for the sharpe tool. The source code exported to the sharpe is simple and intuitively understandable. The procedure includes: (a) define constants, functions and events; (b) decide if an event is basic or repeated; (c) eliminate inconsistencies; (d) build the fault tree and define the measures to be computed.

The functions used in the source code are related to failure and repair CDFs associated with each field device. If we intend to evaluate the network reliability *R*(*t*) then it is only necessary to replace each event related with the failure of device *i* by the respective CDF (as described in Section 4.3), since sharpe computes the device reliability *R_i_*(*t*) from that. However, if we are interested in evaluating the network availability *A*(*t*) then the process is slightly different. If failure and repair rates are constant (*i.e.*, defined by an exponential distribution) then we can use these parameters to compute the availability function of the device *A_i_*(*t*) as described in Section 4.3. Then, it is only necessary to replace each event related with the device *i* by its availability function *A_i_*(*t*) (note: sharpe has already a function that computes this function from the rates). When other CDFs are used to characterize the failure and repair processes (or at least one of them) it is necessary to use the concept of *model hierarchy* provided by sharpe. The reasoning behind this concept is to use the output of one model as the input of another model. Applied to this case, we can model the behavior of device as a semi-Markov chain with two states: *operational* and *failed*. In the former case the device is operational, and in the latter it is failed. Transitions between these two states are described by failure and repair CDFs. As discussed in Section 5.2, these functions must be expressed using exponential polynomial terms. This model can be solved by sharpe and the respective availability (*i.e.*, *operational* state probability) can be used as the input event of the device in the fault tree. Note that it is not necessary to solve this model in advance. The model’s code can be placed in the same code that describes the fault tree, since sharpe analyzes the dependencies between models before computing the results. Details about the use of hierarchical models can be found in [49].

If a device is redundant, sub-events are created according to Figure 7(a); otherwise sub-events are created according to Figure 7(b). An important aspect is to decide if an event is basic or repeated. This aspect is easily analyzed through a search in the basic events of the fault tree. If an event occurs only once, it is considered to be basic; otherwise it is considered to be repeated.

The creation of the fault tree is completely based on discussions presented in Sections 5.4, 5.6 and 5.8. A few precautions should be taken in cases where the logic gates have only one input. This is solved by replacing the logic gate by the input event. Finally, the choice of evaluation function (reliability, unreliability, MTTF, availability, unavailability) or the importance measures (Birnbaum or criticality) is inserted in the source code.

**Figure 13.**
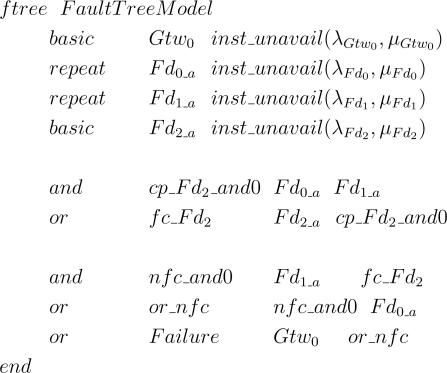
sharpe source code generated by the proposed methodology and based on the fault tree of Figure 12.

An example of generated sharpe source code is presented in Figure 13. This example is based on the fault tree of Figure 12 and assumes that device *i* failure and repair rates are constant, respectively λ*_i_* and *μ_i_*. Note that there are two repeated events, *Fd*_0_*a*_ and *Fd*_1_*a*_. In this example the unavailability function (*inst_unavail*()) was used for the sake of illustration of the functions supported by sharpe. The notation adopted in the source code used to build the fault tree is identical to Figure 12.

## 6. Results

In this section we will present some results obtained when using the proposed methodology to evaluate some dependability metrics in WSN. Our main goal is to highlight some of the capabilities of the proposed methodology, regarding the identification of dependability bottlenecks in WSN, and the capability to evaluate reliability and availability in typical industrial application scenarios. The main assumptions considered in this section are listed below:

- **Scenarios:** we have used line, star and cluster (particular case of mesh topology) topologies.
- **Failure rate:** we assume that device failures occur with a constant rate (*i.e.*, exponential distribution). The gateway and the access point have typically a reliability higher than other network devices. Thus, the gateway and the access point have been configured to have a failure rate with one order of magnitude lower than the field devices and routers. The failure rate of the devices is unknown, however we can use different range of values to measure different behaviors. We assume a MTTF (hours) range to 1 year (λ *≅* = 1e–4), 5 years (λ *≅* = 2e–5) and 10 years (λ *≅* = 1e–5).
- **Repair rate:** similarly, we assume a constant repair rate. Although this could be an unrealistic assumption, it can be proved that this approximation results in small errors if *μ* ≫ λ (which is the case). We assume different range of values for the gateways and access points, when compared with the field devices and routers. The MTTR range for the first two devices was configured to 5 h (*μ* = 0.2) and 10 h (*μ* = 0.1), whereas the MTTR range for the other devices was configured to 1 day (*μ ≅* = 0.04) and 2 days (*μ ≅* = 0.02).

### 6.1. Star Topology

**Figure 14.**
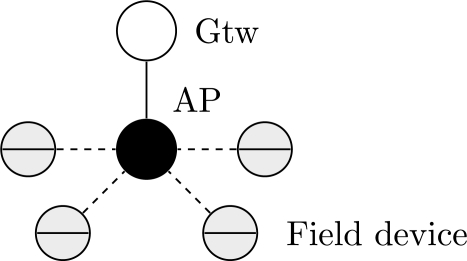
A star topology composed of four field devices.

The first assessed scenario was the star topology. Consider an application that is monitoring the temperature of four boilers. A sensor node is installed in each boiler as described in Figure 14. Within this context, it is assumed that the application fails if at least one field device fails.

**Figure 15.**
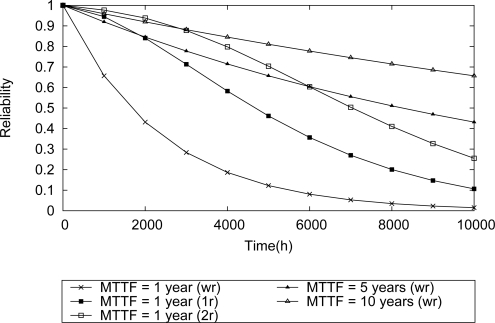
Reliability evaluation for a star topology.

It is intuitive to realize that the network reliability will increase as long as more reliable devices are used. This behavior is shown in Figure 15. If there is no redundancy, the scenario that uses sensor nodes with failure rate of 1E–4 (MTTF—1 year) presents a network reliability lower than all the other scenarios using more reliable field devices.

In what concerns dependability requirements, design decisions are usually related to the selection of more reliable and more expensive devices *vs.* less reliable and inexpensive devices. In general, there is a global policy whose goal is to improve the reliability of the applications. The result presented in Figure 15 can be used for that purpose.

On the other hand, in some cases the application requires an increase of the network reliability, but the acquisition of more robust devices is not an option. A possible solution for this problem is the use of redundancy. For instance, consider the topology described in Figure 14, composed of sensor nodes with failure rate 1E–4. Depending on the number of spare devices that can be used, the network reliability can reach levels comparable with those achieved when more reliable devices are used. According to the results shown in Figure 15, when just one spare device is used (1*r*) in each field device, during 2000 h the WSN achieves reliability levels comparable to the case where field devices five times more reliable are used. If two spare devices are used (2*r*) in each field device, the network reliability during 3500 h presents levels comparable with a scenario where devices ten times more reliable are used. On the other hand, during 6000 h the network reliability has a performance level that is comparable to the case where devices five times more reliable have been used. This result is three times better than the result found when using just single redundancy (1r).

**Figure 16.**
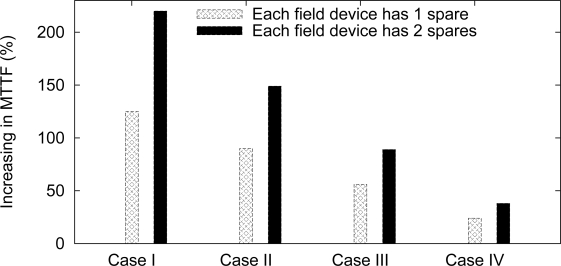
Influence of failure conditions and redundancy levels to the network MTTF.

Another way to evaluate the influence of redundancy is through a MTTF analysis, which is dependent on the network failure conditions. We assume four types of failure conditions: case I, at least one field device fails; case II, at least two field devices fail; case III, at least three field devices fail; case IV, all field devices fail. The results are summarized in Figure 16. For example, if it is considered that the network fails if at least one field device fails, then the use of single redundancy (1r) may increase the network MTTF by 125%, whereas the double redundancy (2r) may increase it by 220%. This MTTF-based analysis can be useful to find the desired application requirements.

### 6.2. Line Topology

**Figure 17.**
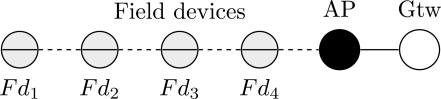
Typical line topology for a WSN.

Line topology is a typical solution used for monitoring pipeline applications. In this case, the information is relayed hop-by-hop until the gateway. Figure 17 illustrates an example of a line topology for a WSN. If a device along the line fails, the monitoring application will also fail.

**Figure 18.**
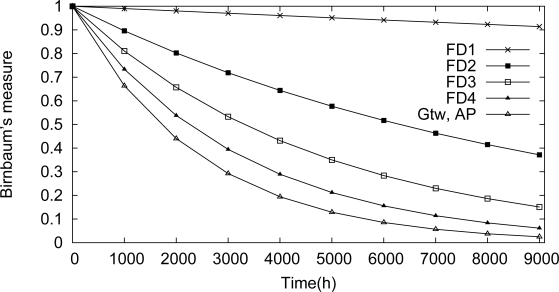
Analysis of the component importance based on Birnbaum measure for the line topology.

**Figure 19.**
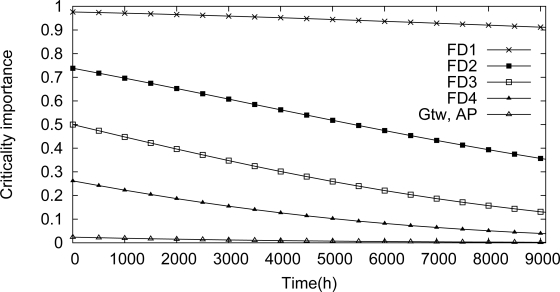
Analysis of the component importance based on Criticality measure for the line topology.

One of the targets of the proposed methodology is to identify dependability bottlenecks in the network. These impairments can be identified through the use of Birnbaum’s measure and criticality importance. A component importance analysis for this scenario is illustrated in Figures 18 and 19. Based on the Birnbaum’s measure (Figure 18), the field device *Fd*_1_ is the device more susceptible to cause a network failure. This behavior is confirmed by the criticality importance, as illustrated in Figure 19. In other words, if the field device *Fd*_1_ fails, there is a high probability that there is no path to the gateway due to a failure in an intermediate device.

Consider a communication scenario where the application requires an improved reliability level and there is just one available spare device. The problem that must be addressed is to select which device should be equipped with the available spare device, so that the reliability level of the system is maximized. A sensitivity analysis for this problem is shown in Table 1. According to the results presented, if the spare device is configured to be at the gateway or access point, the MTTF of network will be increased by around 2%. Thus, there is no real advantage to configure two spare devices in the gateway or in the access points. The best result, as expected, is attained when the spare device is configured to be at device *Fd*_1_. In this configuration, the network MTTF is increased by 19.23%. This configuration presents even better performance than the configuration with two spare devices in *Fd*_3_ or *Fd*_4_. Note that if the spare device have been configured in *Fd*_4_, the network MTTF would have been decreased by 1.95%.

### 6.3. Cluster Topology

**Figure 20.**
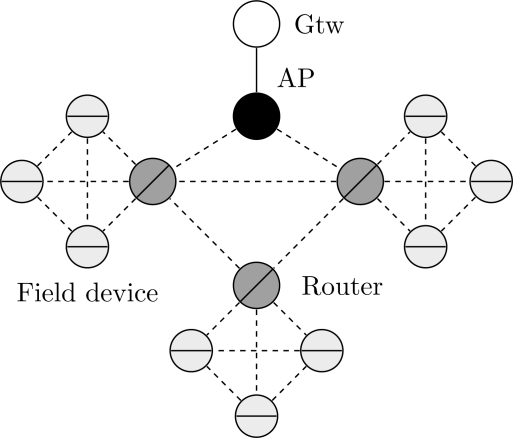
Typical cluster topology for a Wireless Sensor Network.

In general, a cluster topology is used when there is the need to segregate partially a network. Each cluster may assume specific tasks, for example, monitoring a region, traffic prioritization, provide redundancy, *etc*. Figure 20 illustrates a typical cluster topology for a WSN, where the clusters communicate each other through router devices.

Consider for example an application where each cluster monitors an industrial control loop. The application will fail if at least one of the cluster fails. On the other hand, a cluster will fail if all field devices within the cluster fail. The dependability target is to maximize the availability of the application and consequently to minimize the application outage (measured in hours per year).

**Figure 21.**
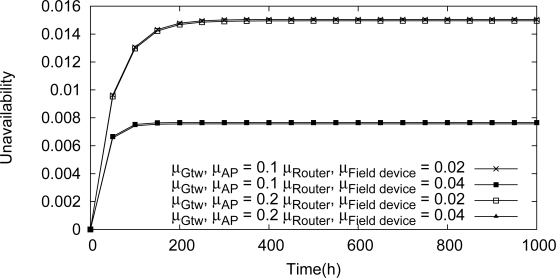
Influence of the maintenance of devices in the network unavailability.

We have analyzed the influence upon the network unavailability of maintenance operations. The results of this analysis are illustrated in Figure 21. It can be observed that changes in the repair rate of the gateway or the access point do not cause significant changes in the network unavailability. On the other hand, the repair rates of the field devices and the routers have strong influence on the network unavailability. If, for example, the repair rate of one these devices is doubled, the application outage is decreased by around 50% (131 hours per year to 66 hours per year).

**Figure 22.**
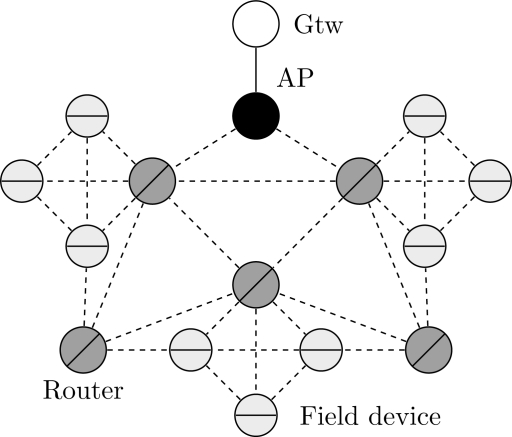
Adding router devices to improve reliability and availability.

According to the results presented in Figure 20, a critical device is the head of cluster. This function is executed by a router device. Assuming that there are two spare router devices available, the bottleneck of cluster can be minimized in two different ways: configuring a structural redundancy (scenario illustrated in Figure 20) for two router devices where each one receives a spare, or configuring the two spare devices as new router devices in the network as illustrated in Figure 22. The results from this analysis are presented in Table 2. In the first case, when redundancy has been used, the application outage time was around tree times more efficient than the scenario presented in Figure 20 (*μ_gtw_*, *μ_AP_* = 0.2 and *μ_router_*, *μ_field device_* = 0.02). On the other hand, the use of two new routers can significantly decrease the application outage by around 65 times. This result is due to the creation of new paths to the gateway, when new router devices are added to the network.

## 7. Conclusions

In this paper we have proposed a methodology to evaluate the dependability of Wireless Sensor Networks in typical industrial environments. For this purpose, we have modeled a WSN using a fault tree-based formalism, considering permanent faults that occur in field devices due to hardware problems and the absence of routes to the gateway.

To validate the proposed methodology, we select several scenarios commonly found in industrial applications. The results obtained show that the proposal is useful to identify dependability bottlenecks, to estimate the required redundancy level and to aid the design throughout the life cycle of the network.

In future works we intend to support unreliable link, thus considering transient failures. Furthermore we also intend to consider the coverage factor related with the reconfiguration mechanisms, and also common-cause failures.

## Figures and Tables

**Table 1.** Sensitivity analysis.

**Device**	**Increase in MTTF**	
	**1 spare device**	**2 spare devices**
Gtw	2.32%	2.43%
AP	1.98%	2.40%
*Fd*_4_	−1.95%	14.05%
*Fd*_3_	3.60%	17.19%
*Fd*_2_	10.44%	20.90%
*Fd*_1_	19.23%	25.43%

**Table 2.** Means to improve the availability of network.

**Scenario**	**Unavailability**	**Outage (hours per year)**
Normal	0.015	131
Repair 2× more fast	0.007	66
Redundancy	0.005	44
2 new routers	0.00025	2
